# The Institutional Worker

**Published:** 1920-10-30

**Authors:** 


THE
### INSTITUTIONAL WORKER
**Being a Special Supplement to "The Hospital"**

### OUR BUREAU OF INFORMATION.

### Rules for Correspondents

1. Every letter must be accompanied by the coupon to be cut from the back cover (inside page) of The Hospital, current issue, and must contain the name and address of the correspondent with pseudonym for publication if desired. Replies by post cannot be given save under exceptional circumstances at the Editor's discretion.

2. Letters from Approved Homes in reply to special needs published in the Bureau should state terms and full particulars, and be sent prepaid under cover to the Editor of the Bureau with name written across coupon for identification.

3. Proprietors of Homes which have not yet been entered on the List of Approved Homes, but have spare accommodation likely to suit special needs, are invited to write for an application form for registration. The fee for registration, which includes two announcements of the Home in the Bureau and other privileges, is 10s.

4. All communications to be addressed to the Editor of The Hospital, 28 Southampton Street, Strand. London, W.C. 2, and marked "Bureau of Information."

### INSTITUTION FACTS AND FIGURES.

### THE COOKING OF DRIED FRUITS AND VEGETABLES.

To the practical housekeeper the problem of cooking fruits and vegetables, which have been dried for later consumption, in such a way as to bring out their flavour and make them both attractive and wholesome, is full of interest and, not infrequently, of anxiety. In an article published in The Hospital of October 16, p. 50, the preparation and storage of these foods were discussed and the result of certain investigations described. The United States Department of Agriculture has been making exhaustive experiments and inquiries in connection with a further stage in the preparation of dried fruit and vegetables—i.e., their cooking—and *The Modern Hospital* (Chicago) publishes a progress report, full of interest and instruction, from the experimental kitchen of the Office of Home Economics, Washington.

The first point for housekeepers to remember concerning dried vegetables is not to keep them too long, for, though they do not spoil, the colour, flavour, and cooking qualities do not remain indefinitely. It is desirable, therefore, to use them in the early part of the winter. They may be soaked from one to three hours in warm or cold water, and then cooked from ten to thirty minutes, or longer if required. If not soaked they should be put into boiling water and allowed to simmer slowly from twenty to forty minutes or longer. The quantity of water to be used for the soaking and cooking is important. It is rightly insisted—and we could wish this point were realised by English cooks in regard to fresh vegetables—that to drown vegetables in a large amount of water, which is then thrown away, is the most thorough method which could be devised for extracting and discarding not only the flavour but the nutritive constituents. The water added for soaking or cooking should be little more than the vegetables will take up. A good general rule is three cups of water to one of vegetable; but half that amount of water in the case of spinach and greens, which are light in volume. It is better to salt dried vegetables towards the end of the cooking process rather than at the beginning.

As soon as the flavour of dried vegetables is noticed to be deteriorating, special care should be taken in the service for table of the remaining stock. If they are then served in the usual way—i.e., after being plain boiled in water—the housekeeper need not be surprised if she finds the plates contain large "leavings." This is where the good cook shines, and if she has a genius in this direction, or if only she takes a little pains, she will find that dried vegetables which may be getting somewhat stale can be, in combination with fresh vegetables, or other materials, so used as to become an appetising and attractive dish. Of course all dried vegetables must first be made tender by soaking and boiling; but there is no end to the ways of serving them either as a *purée*, with other vegetables in a hot pot, as a salad, stewed or braised with some meat stock, with cheese, or converted into soups.

### Dried Fruits.

Here again care should be taken not to soak or cook in too much water. Equal measure of water and fruit is recommended and the cooking done slowly and long in water below the boiling-point. Dried fruits may be soaked for a few hours or put directly, after washing, into the warm water for cooking.

### THE HOSPITAL MORTUARY.

### Responsibility for Burial.

A hospital is not bound to have a mortuary attached to it; it is, however, essential that some provision should be made for the immediate removal of the body. This might be arranged through a local undertaker, some of whom have private mortuaries. Experience proves, however, that an arrangement of this character is never entirely satisfactory, and that sooner or later the hospital will suffer inconvenience. Consideration for the patients' friends and their convenience ought not to be lost sight of, and on these grounds alone it is highly desirable that every hospital should be provided with a mortuary. It is the duty of the executors of the deceased to provide for the burial of the body, but though they are primarily responsible, it is also a duty imposed by law on the householder of any house where death occurs to see that notice of such death is given to the parish authorities. If the deceased be a pauper, the Guardians or overseers have power to bury the body at the expense of the parish. Doubtless, if it could be shown that the presence of the dead body in the house or hospital pending the time needful for arranging the burial is a source of danger to health or a nuisance to others, the local health authority will arrange for the transference of the body to the public mortuary or some suitable place.—E. W.

### Substitute for Lead Paint.

Paripan, Ltd., of Sherwood House, Piccadilly Circus, London, W. 1, supply what is known as Parax Paint—a paint which takes the place of lead paint. Paripan, Ltd., hold that outside work will last much longer and look better if Parax Paint is used, as it provides better protection than the lead. We suggest that you write to Messrs. Paripan for prices and a sample or specimen of the paint.—K. W.

### Question for October.

### PATIENTS' WAITING-LISTS.

**Describe a simple method of recording names of patients awaiting admission, showing at a glance their proper turn for admission having regard both to period of waiting and the medical urgency of their respective cases.**

**(A minimum payment of 10s. 6d. will be made for each published answer.)**

RULES.

The following rules must be observed :—

1. Contributions must be written on one side of the paper. Conciseness and terseness are desirable features. The MS. must bear the name and address of the sender and be accompanied by coupon to be cut from the back cover (inside page) of the current issue of THE HOSPITAL. A pseudonym must be chosen if the name is not to be published.

2. Contributions must be addressed to the Editor of THE HOSPITAL Institutional Worker Supplement, 28 & 29 Southampton Street, Strand, London, W.C. 2, should reach him before the end of the current month, and be marked in the left-hand corner "Facts and Figures."

### QUESTIONS INVITED.

IN connection with our Question Box, we offer every month five shillings each for the two best questions which are sent in for consideration. The questions may be on any institutional subject and concern any department, from the secretary's to the porter's, or the matron's to the domestic staff; the one essential is that they must be practical and deal with points that have a definite relation to hospital or institutional administration, upkeep, finance, or management. Points relating to artificers' work, housekeeping, the laundry, out-patients, and other practical matters will be welcome. It is hoped that thus, when difficult or doubtful points arise in the course of their work, institutional workers will be encouraged to put them in the form of a question and send them to the Editor, THE HOSPITAL Bureau of Information, 28 Southampton Street, Strand, W.C. 2, marked "Question Box."

The best questions will be published in due course and our readers will be given the opportunity of answering them. Thus all institutional workers may be able to co-operate with the view of helping the work and smoothing the difficulties of each other. In short, we want the Question Box of the *Institutional Worker* to be freely used by every worker habitually.

**The Editor will be glad to receive correspondence and to consider contributions upon all subjects relating to institutional work which affect the welfare of institutional workers.**

### ENQUIRIES AND ANSWERS.

### *THE SICK AND IN NEED.*

### Help for Demobilised Nurse.

YOU might write to the Nurses' Resettlement Committee, 112 George Street, Edinburgh, and ask if they can help you to obtain some suitable work. The only other alternative we can suggest is that you reply to advertisements that appeal to you and which appear in all nursing journals.—AILSA.

### Address of Nurses' Resettlement Department.

THE address of the Nurses' Resettlement Department in London is 99 Queen's Gate, S.W. 7. See reply to "Ailsa."—C. A. C.

### *EMPLOYMENT AND TRAINING.*

### Massage School in or Near Glasgow.

THERE is a school of massage attached to the Western Infirmary, Glasgow. Write for full particulars to the Matron. The Secretary of the C.S.M.M.G. (I.S.T.M.), 151 Great Portland Street, London, W. 1, will forward you addresses of other massage schools upon application. Enclose a stamped addressed envelope for reply.—ELSIE.

### Lectures for Health Visitors in Liverpool.

WE suggest that you write to the Hon. Secretary, Public Health Department, Municipal Offices, Dale Street, Liverpool, and ask for particulars and prospectus of lectures which are to be given during the 1920-21 session. The address of the University School of Hygiene is Mount Pleasant, Liverpool.—DOROTHY.

### *PRACTICAL MATTERS.*

### Half-Watt Lamps.

IT is certainly in some circumstances economical to make use of half-watt lamps, but if you wish to economise current consumption you must take into consideration when replacing lamps with this type what amount of current the old lamps consumed. For instance, an ordinary sixteen-candlepower metal-filament lamp consumes 30 watts. It would not pay you to put in place of this a half-watt lamp consuming 60 watts and giving 120 candlepower light, unless, of course, additional light were required. The term half-watt implies that the lamp consumes half a watt for every candle-power of light, as against nearly one watt of candle-power consumed by the ordinary lamp. In low voltages (from 20 to 30) half-watt lamps can be obtained at a candle-power consuming a minimum of 15 watts, but for voltages of 100 and over the smallest lamp consumes 60 watts. The prices range from 4*s.* 3*d.* each, less a trade discount ordinarily allowed by association makers. It is therefore nearly double the price of the ordinary type of lamp. The average life of both types is about 1,000 hours.—J. P.

### *MISCELLANEOUS.*

### Electric Lighting Units.

PROBABLY you could not do better than apply to Messrs. Crossley Bros., Ltd., of 139 Queen Victoria Street, E.C. 4, for particulars of electric lighting and power units. This firm has a large experience of work of this character.—K. M. C.

### Pensions Payment to Out-patient Clinics.

THE payments made by the Ministry of Pensions in respect of orthopædic clinics is 1s. 6d. an attendance, not including medical supervision, or 2s. including medical supervision. For special medical and surgical clinics (including cardiac, tropical disease, aural and ophthalmic) a payment of 3s. per attendance is allowed, which includes the services of medical or surgical specialists.—K. L. C.

### Powers of Local Authorities in Infectious Diseases.

WE should advise you to read Chapter VI., Vol. III., of "Hospitals and Asylums of the World," by Sir Henry Burdett, K.C.B., pp. 102 *et seq.*, dealing with Infectious Disease Hospitals in the Provinces. You should further procure the following Acts: Infectious Diseases Prevention Act, 1855; Sanitary Act, 1866; Public Health Act, 1875 (the greatest advances have been made under this Act); the Infectious Disease (Notification) Act, 1889; Infectious Diseases (Prevention) Act, 1890; and the more recent Infectious Disease Notification, etc., Acts.—E. L.

### The "Aurorascope."

THE "Aurorascope" you refer to can be obtained from the "Aurorascope" Company, Ltd., Fulwood House, High Holborn, London, W.C. 1, or from any surgical instrument maker. The price of the best quality outfit is £2 2s. This instrument gives illumination of the ear, mouth, throat, and teeth, and can also be used for trans-illumination of the antrum and retinoscopy. No dark room or head-lamp is required.—F.R.C.P.

### High-Frequency Apparatus.

IT is possible to obtain a high-frequency violet-ray generator for £15 15s. The "Stirling" high-frequency violet-ray generator, obtainable of the Stirling Corporation, Ltd., 16 Wigmore Street, London, W. 1, at the price mentioned can be operated without any special wiring, from any lamp or wall plug, and works equally well with direct or alternating current. It is made for two ranges of voltage, one 90-150 volts and the other 190-250 volts. The whole outfit, with carrying case, measures $10\frac{1}{2}$ inches by 8 inches by 4 inches, and weighs only $5\frac{1}{2}$ lb. A battery-type generator is supplied by the company in cases where the electric current is not available.—F. S.

